# Using content validity index methodology for cross-cultural translation of a patient-reported outcome measure for head and neck cancer

**DOI:** 10.3389/frhs.2025.1582127

**Published:** 2025-06-20

**Authors:** Janet H. Van Cleave, Alizendie Guerra, Eva Liang, Carolina Gutiérrez, Ron J. Karni, Marcely Tsikis, Geanise Pearl C. Nguyen, Allison P. Squires

**Affiliations:** ^1^ Department of Nursing Research, UTHealth Houston Cizik School of Nursing, Houston, TX, United States; ^2^ NYU Meyers College of Nursing, New York, NY, United States; ^3^ Office of Research and Innovation, Stony Brook School of Nursing, Stony Brook, NY, United States; ^4^ Department of Physical Medicine and Rehabilitation, UTHealth Houston, McGovern Medical School, Houston, TX, United States; ^5^ Department of Otorhinolaryngology–Head & Neck Surgery, UTHealth Houston, McGovern Medical School, Houston, TX, United States

**Keywords:** limited english proficiency, immigrants, patient reported outcome measures, head and neck cancer, public health

## Abstract

**Introduction:**

Translations of patient-reported measures may not account for structural and cultural differences in shared languages spoken in multiple countries, such as English, Spanish, Arabic, or Russian. The objective of this research was to create a cross-cultural Spanish translation of the New York University (NYU) Electronic Patient Visit Assessment (ePVA)^©^ for head and neck cancer (HNC), a patient-reported symptom measure available only in English.

**Methods:**

Using the Content Validity Index (CVI) methodology, an expert panel of nurses (*n* = 6) proficient in Spanish and English independently reviewed and rated a forward translation of the ePVA, a measure consisting of 21 categories of symptoms common to HNC. The panel rated the cultural relevance (1 = not relevant, 2 = somewhat relevant, 3 = very relevant, 4 = highly relevant) and translation equivalence (1 = yes or 0 = no) of each ePVA item. The CVI cultural relevance and translation equivalence scores for each item (item CVI) were calculated as the proportion of experts agreeing that the item was very relevant or highly relevant and the translation was equivalent. The scale CVI score was an average of the item CVI scores; the minimum accepted scale CVI score was .80. Items with CVI scores <0.59 were labeled as problematic items and evaluated through cognitive interviews with native Spanish-speaking patients (*N* = 4) diagnosed with HNC.

**Results:**

The translation was acceptable in cultural relevance (average CVI score = 0.95) and equivalence (average CVI score = 0.84). Cognitive interviews revealed 9 problematic items that differed in words and meaning, primarily addressing pain and swallowing symptoms. These items were refined and included in the final translation of the Spanish ePVA.

**Conclusion:**

These study findings underscore the need for survey instrument translations that account for variations in shared languages spoken across countries.

## 1 Introduction

More than 25 million residents of the United States (US) have limited English proficiency (LEP) (1, 2), defined as individuals who do not speak English as their primary language and have a limited ability to read, write, or understand the English language (1, 3). These communication barriers for those with LEP have healthcare consequences. Those with LEP often experience longer hospital stays and unplanned emergency room revisits within 72 h after hospital discharge because of difficulty understanding hospital discharge instructions and obtaining prescriptions (1, 4). In cancer care, patients with LEP may face additional barriers to care. Research has demonstrated that those with LEP and diagnosed with cancer have lower rates of clinical trial engagement and patient portal messaging than English-speaking patients (5). Use of patient-reported symptom monitoring (PROM) may overcome communication barriers for patients with LEP. PROMs in routine cancer care are associated with increased symptom control and decreased emergency room and urgent care visits (6–11).

PROMs can be an important tool of management for patients with head and neck cancer (HNC) because of the high symptom burden experienced by the HNC population. HNC is the 6th most common cancer worldwide (12). Over 72,000 individuals in the US and 947,000 worldwide are diagnosed with HNC annually (13, 14). HNC comprises tumors that arise from the lip, oral cavity, pharynx, larynx, and paranasal sinuses. The most common risk factors for HNC are tobacco and alcohol use disorders**.** Other risk factors are exposure to human papillomavirus (HPV)**,** Epstein–Barr Virus**,** and Betel nut chewing, which is common in Asian countries (15–19)^.^ HNC is more likely to occur in men than women (Men: 72.4%; Women: 27.6%) (13).

In the US, most patients are treated aggressively with multimodal therapies (surgery, chemotherapy, radiation therapy, and immunotherapy). This aggressive treatment has contributed to an increased 5-year survival rate from 60%–68% over the past 20 years (20), expanding the number of survivors with HNC to 540,150 in 2025 (21). The increased survival in HNC comes with a human cost; up to 60% of survivors with HNC experience substantial symptom burden (e.g., severe difficulty swallowing, chronic pain) and functional morbidity (e.g., limitations in movement and inability to work) (22–24).

Quality care for patients with HNC includes close systematic monitoring of patients' symptoms and functional morbidity, enabling clinicians to make early, real-time interventions that optimize patient outcomes (25–28). Thus, our team explored implementing a PROM in HNC clinical care. The initial step in this project was to conduct a systematic literature review of symptom and function measures to identify valid and reliable PROMs that patients can answer in approximately 10 min while waiting for their oncology visits. This literature review showed that the existing valid and reliable instruments have properties that limit their use in routine clinical practice. Many instruments were lengthy, complicated, and intended for general cancer populations, which posed an unneeded respondent burden for patients with HNC. Some instruments contained *a priori* assumptions on associations between specific symptoms and functional morbidity (e.g., “I cannot eat because of pain”), potentially introducing unintentional bias into data. Moreover, the existing instruments did not fully capture the long-term symptom experience and functional morbidity after treatment completion experienced by the growing number of patients living longer with HNC. Hence, we developed a patient-centered PROM, New York University (NYU) Electronic Patient Visit Assessment (ePVA)^©^, using rigorous measurement theory including content validity analysis. Subsequent testing of the ePVA has provided evidence of its reliability and convergent validity with health-related quality of life (HRQoL), patient and provider acceptance, and an association with clinically significant differences in patients' symptoms and acute care use (29–31).

Like many measures used as PROMs, the ePVA was developed in English, which impedes its use among patients with LEP (32). The team decided to translate the ePVA from English to Spanish to improve communication with the Hispanic immigrants with LEP. A challenge with the translation process was that the Hispanic population in the US constitutes a diverse population with origins in multiple states and countries, including Puerto Rico, Central America, and South America (33). To overcome this challenge, the team used the Content Validity Index (CVI) methodology, a comprehensive and systematic approach to translation that accounts for cross-cultural differences in shared languages.

The CVI methodology is an approach derived from measurement theory and is useful for determining consensus among experts (34–36). Squires et al. (37) expanded the CVI methodology for use in cross-cultural translations of measures to overcome limitations in existing guidelines that focused on technical aspects of cross-cultural translation, had limited content expert feedback, and did not use a systematic method of identifying problematic items requiring further refinement (37). The expansion of the CVI methodology incorporated a systematic process of independent analysis that includes content expert input of qualitative data to assess the back translation, analysis of cultural relevance and translation equivalence, and identification of problematic survey items that require further refinement (37). The CVI methodology has been used to develop and validate a survey for RN4CAST, a comparative nursing workforce study conducted in 12 European countries using 11 languages with modifications for regional dialects (37), and multiple workforce assessment studies (37–44).

The objective of this article is to describe our experience using the CVI methodology in translating the ePVA from English to Spanish (i.e., the Spanish ePVA). This article will present the CVI methodology used to create the Spanish ePVA, analyze the quality of the Spanish ePVA translation, and discuss the benefits and limitations of the CVI methodology in translating PROMs. The study's specific aims are to (1) Produce a Spanish ePVA with acceptable cultural relevance and translation equivalence using CVI methodology and (2) Evaluate and refine problematic items of the Spanish ePVA using cognitive interviews.

## 2 Methods

### 2.1 Study design

The translation of the ePVA from English to Spanish was accomplished using a comprehensive and systematic approach guided by the CVI methodology. This approach encompasses recommended good practices in the translation of PROMs by using experts to evaluate the forward and back translations, using ratings to identify problematic items, and conducting interviews with the targeted population to refine the translated PROMs (35, 45). Further, this method evaluates the cultural relevance and translation equivalence of the individual Spanish ePVA items and average scale CVI.

### 2.2 NYU electronic patient visit assessment (ePVA)^©^ for HNC

The ePVA's development, reliability, and clinical usefulness in symptom monitoring for patients with HNC have been described elsewhere (29, 30, 46). The ePVA is a PROM consisting of 21 categories of symptoms common to HNC (i.e., pain, eye, ear, nasal, mouth, voice, fibrosis, edema, skin, gastrointestinal, fatigue, movement limitation, sleep, breathing, difficulty eating or drinking, swallowing, communication, social activities, anxiety, depression, and daily activities). The ePVA's design was based on the Theory of Unpleasant Symptoms (47), a middle-range theory that conceptually maps the link between symptoms and outcomes such as HRQoL (29, 30). The measure consists of binomial questions (yes = 1/no = 0) to indicate the presence or absence of the symptom. Some questions are conditional, with branching logic to tailor the assessment to the patient's health status and limit the respondent burden. For example, one question asks if the patient has experienced pain in the past 7 days. If the patient answers “yes,” the ePVA directs the patient to complete a multidimensional pain assessment. If the patient responds “no,” the ePVA leads the patient to answer questions about a different symptom. Prior research has shown that the ePVA measure has acceptable reliability using the Kuder-Richardson Formula 20 measure for questionnaires with binary variables (alpha = .82 -.85) and convergent validity with HRQoL (29, 30, 46, 48).

### 2.3 Translation procedures

**Figure 1:**
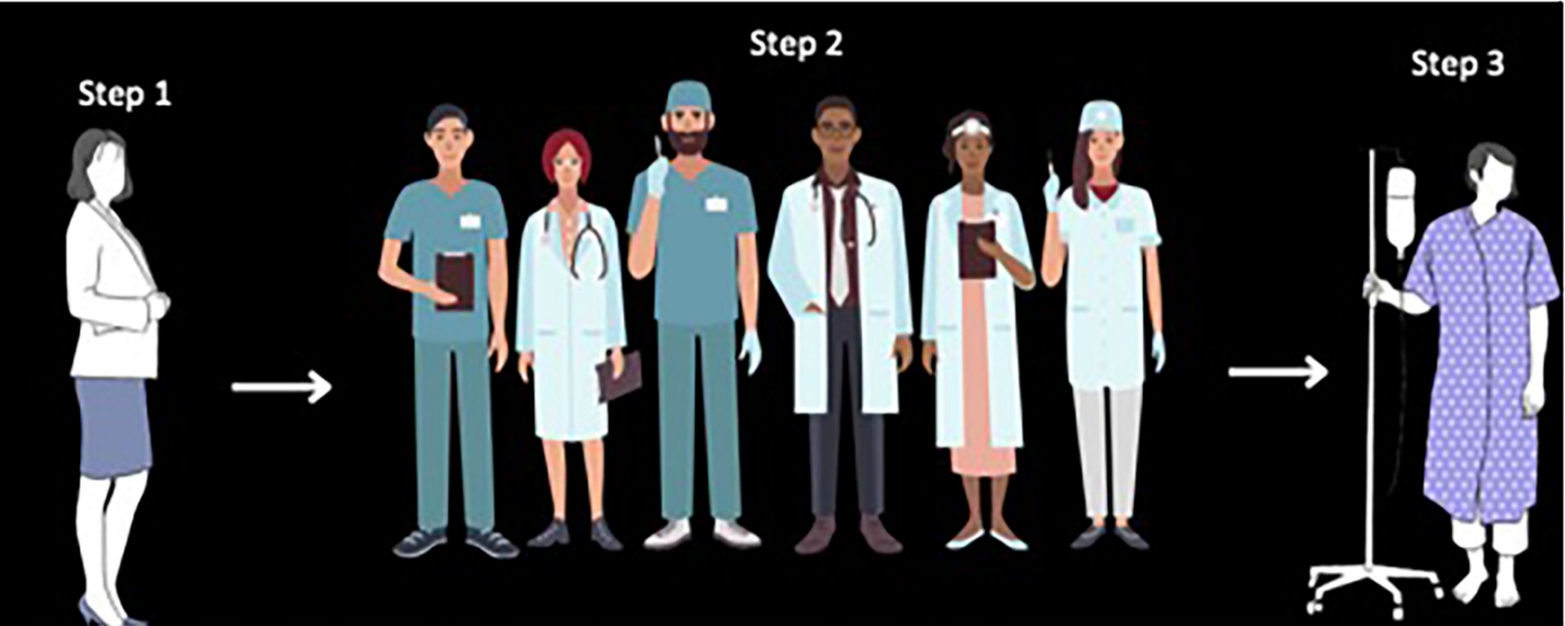
Translation process of the Spanish NYU electronic patient visit assessment (ePVA) for head and neck cancer. The content validity index (CVI) methodology consisted of a three-step process. Step 1—Forward Translation: The senior author (AS), fluent in Spanish and English with expertise in language discordance and its effect on healthcare outcomes and use of CVI for cross-cultural translations of surveys, completed a forward translation of the ePVA. Step 2—Independent Review and Rating of Forward Translation: An expert panel (*N* = 6) fluent in Spanish and English and qualified as a nurse to understand and interpret medical terminology independently reviewed and rated a forward translation of the ePVA. The panel rated relevance (1 = not relevant, 2 = somewhat relevant, 3 = very relevant, 4 = highly relevant) and translation equivalence (1 = Yes or 0 = No) of each ePVA item. The panel members also recorded their recommendations when they disagreed with the relevance or translation equivalence of the forward translation of the Spanish ePVA, thus providing qualitative data on the back translation of the Spanish ePVA. The item-CVI relevance and translation equivalence scores were calculated as the proportion of experts agreeing that the item was “very” or “highly” relevant and the translation was equivalent. Items with CVI scores of <0.59 were labeled as potentially problematic items. Step 3: The team conducted cognitive interviews with native Spanish-speaking patients (*N* = 4) with head and neck cancer to evaluate potentially problematic items.

The translation process of the Spanish ePVA consisted of forward translation, expert panel independent review and rating of the forward and back translation, and cognitive interviews with native Spanish-speaking patients (*n* = 4) with HNC to evaluate problematic items (See Figure 1).

#### 2.3.1 Forward translation

The senior author (AS), fluent in English and Spanish with expertise in language discordance and its effect on healthcare outcomes and the use of CVI for cross-cultural translations of surveys, conducted the forward translation of each ePVA question stem and response option to Spanish, totaling 368 items. The study team entered the English ePVA and the corresponding forward translation in Spanish as a survey in the Research Electronic Data Capture (REDCap) database, a secure web-based software platform designed to support data capture for research studies (49, 50).

#### 2.3.2 Expert panel independent review and rating of the forward and back translation of the ePVA

The research team recruited content experts from across the US to independently review and rate the forward and back translation of the ePVA by issuing a call across the US through a national nursing organization. The requirements to serve on the expert panel were fluency in English and Spanish and qualified as a nurse to understand and interpret medical terminology. Six persons responded to the call; the regions that influenced the panel members' Spanish proficiency were Mexico (*n* = 2), South America (*n* = 2), or not reported (*n* = 2). The panel independently compared the English and Spanish versions of the ePVA for the cultural relevance and translation equivalence of each item by rating cultural relevance using a 4-point Likert scale (1 = not relevant, 2 = somewhat relevant, 3 = very relevant, 4 = highly relevant) and translation equivalence (1 = yes, 0 = no). Panel members also entered qualitative data in text fields to record their recommendations when they disagreed with the cultural relevance or translation equivalence of the forward translation of the Spanish ePVA.

##### 2.3.2.1 Analysis of the quality of the Spanish ePVA translation

The analysis of the quality of the Spanish ePVA translation was conducted using CVI values at the item and scale levels. A CVI value was computed for each item's cultural relevance and translation equivalence (i.e., item-level CVI or I-CVI). The I-CVI for cultural relevance was computed as the proportion of the expert panel in agreement that the item was very relevant or highly relevant (36, 37). The proportion's numerator was the number of experts rating the item as either “3” or “4,” and the denominator was the number of experts completing the assessment. The I-CVI for translation equivalence was computed as the proportion of experts rating the item as having translation equivalence. The proportion's numerator was the number of experts rating the item as “1,” and the denominator was the number of experts completing the assessment. The measure's scale-level CVI (i.e., S-CVI) was calculated as the average of all I-CVIs (i.e., S-CVI/Ave). The minimally acceptable value of translation quality was S-CVI/Ave = 0.80 (51), and the goal was S-CVI/Ave ≥ 0.90 (36).

##### 2.3.2.2 Using modified kappa scores to identify problematic items

Each item's modified kappa score (mKappa) was calculated to adjust the I-CVI score for chance agreement among panel members. This calculation incorporates the probability of chance agreement (Pc), using the equation Pc = [N!/A! (N–A)!] × 0.5 N, where N is the number of experts and A is the number of experts who agree the item was relevant (scores 3 and 4) (35, 36). The probability of chance agreement or disagreement (i.e., mKappa) was calculated as mKappa = [I-CVI-Pc]/[1-Pc] (36). The items with mKappa scores <0.59 were categorized as problematic items, requiring further review using cognitive interviews (36).

#### 2.3.3 Cognitive interviews

The study team conducted cognitive interviews with patients who were native Spanish-speaking patients diagnosed with HNC regarding the problematic items. The methodology described by Knafl et al. (52) guided the construction of the semi-structured interview guide, procedures, and analysis to explore patients' understanding and interpretation of the problematic items (See Supplementary A) (52). The patient eligibility criteria for the cognitive interviews were individuals 18 years and older with histologically diagnosed HNC, fluent in Spanish, and could provide informed consent. The study team recruited all race, ethnicity, and genders before, during, or after HNC treatment (i.e., surgery, chemotherapy, radiation therapy, or a combination of these modalities).

**Figure 2:**
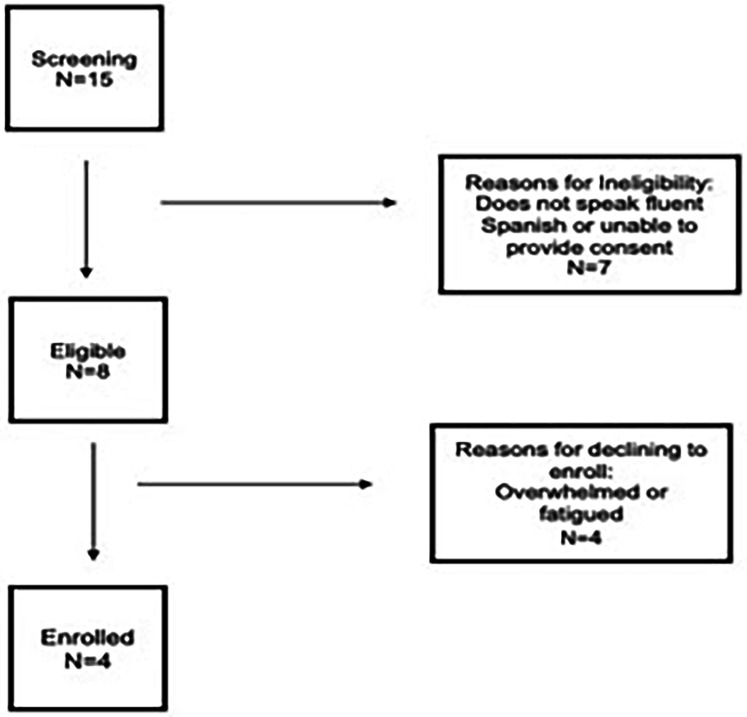
Participant recruitment and enrollment for cognitive interviews.

Ultimately, four native Spanish-speaking partients were enrolled in the study. The participants' demographic data were obtained from both participants and the participants' electronic health records (See Figure 2). After the participant provided informed consent, a study team member fluent in Spanish and English read each question to the participants and recorded the participants' responses on a Microsoft Excel spreadsheet. After eliciting similar answers from three consecutive participants, the team stopped conducting cognitive interviews.

##### 2.3.3.1 Cognitive interviews analysis

After completing the cognitive interviews, team members analyzed a summary table of the cognitive interviews (52). This analysis systematically compared items' mKappa scores, the expert panels' qualitative data on the Spanish ePVA's back translation, and participants' responses to the cognitive interviews to identify the problematic aspects of the items. The team discussed all items until they achieved consensus on retaining, deleting, or modifying the items for the final translation of the Spanish ePVA. Memos of discussions and decisions ensured the trustworthiness of the analysis. The final version of the Spanish ePVA underwent beta testing by nurses and patients with HNC whose Hispanic heritage included Puerto Rico, Central America, and South America.

## 3 Results

### 3.1 Aim 1: produce a Spanish ePVA with acceptable cultural relevance and translation equivalence using CVI methodology

The quality of the Spanish translation of the ePVA was evaluated using S-CVI/Ave scores of cultural relevance and translation equivalence. The analysis found the S-CVI/Ave score for cultural relevance was 0.95, meeting the team's goal criteria of S-CVI/Ave of ≥0.90. A review of individual items' mKappa scores for cultural relevance found only two items with a rating of <0.59, indicating these items as problematic. A further review of the items revealed grammatical errors in the forward translation. The team corrected the errors and included the refined items in the Spanish ePVA.

The S-CVI/Ave for translation equivalence was 0.84, meeting the minimally acceptable value of S-CVI/Ave = .80. A review of individual items' mKappa scores for translation equivalence revealed 55 items with a rating of <0.59, indicating these items as problematic. A further review of these items revealed that 31 of the 55 problematic items had typographical or grammatical errors in the forward translation. The team corrected these errors and included these items in the Spanish ePVA. The remaining 24 problematic items were further analyzed using cognitive interviews.

### 3.2 Aim 2: evaluate and refine problematic items of the Spanish ePVA using cognitive interviews

The team conducted cognitive interviews with patients with HNC (*n* = 4) whose native language was Spanish to evaluate the remaining 24 problematic items. The participants were men with a median age of 61.5 (range 56–70) (See Table 1). The origin country influencing their Spanish language was Puerto Rico (*n* = 2) or not reported (*n* = 2). All participants were undergoing chemotherapy with or without radiation therapy. After informed consent, the participants answered questions followed by probes (See Supplementary A – Cognitive Interview Probes). The analysis of the cognitive interviews revealed two key problems with the 24 items—(1) different words or grammatical structures representing similar concepts or meanings, and (2) different words or grammatical structures representing different concepts or meanings across cultures.

**Table 1:** Demographic characteristics of patient participants for cognitive interviews (*n* = 4).

Variable	Median (Range)
Age	61.5 (range 56–70)
Variable	N (%)
Gender	
Male	4 (100)
Female	0 (0)
Race	
White/Other	4 (100)
African/American	0 (0)
Country of Origin	
Puerto Rico	2 (50)
Unidentified	2 (50)

#### 3.2.1 Different words or grammatical structures representing similar concepts or meanings

The analysis found that 15 of the 24 problematic items differed in words or grammatical structure but represented similar concepts or meanings to participants (See Table 2). These items included instructions for answering the ePVA questionnaire and the following symptoms: pain, skin, eye, mouth, gastrointestinal, voice, movement, breathing, social function, and swallowing. For example, the term itchy skin had alternate translations of “picazones,” “picazón,” or “picazon la piel,” yet the participants considered the different translations as equivalent. The team discussed and refined all items to account for cross-cultural variations and included the items in the final translation of the Spanish ePVA.

**Table 2:** Spanish ePVA items with different words or grammatical structures representing similar concepts or meanings.

English	Item Description	Forward Translation	Suggested Alternate Translations By Expert Panel and Patient Participants	Back Translation	Final Version in Spanish ePVA
Please Point to Places on the Figure Closest to Where You Have Pain (Check All That Apply)	Instructions	Toca las partes de la figura en la pantalla donde Ud. tiene dolor. (Marque toda que aplica)	Por favor toque las partes de la figura en la pantalla donde Ud. tiene dolor. (Marque todo lo que aplica)	Please touch the parts of the figure on the screen where you have pain. (Check all that apply)	Question was deleted because figure was not included in final version
			Señale en la figura los lugares más cercanos a donde siente dolor. (Marque todas las que apliquen)	Mark in the figure the places closest to where you feel pain. (Check all that apply)	
In the past 7 days, I had pain	Pain Symptoms	En las ultimas 7 días, hube tenido dolor.	he tenido dolor	I have had pain	En los ultimos 7 dias, tuve dolor
			En los últimos 7 días, he tenido dolor	In the past 7 days, I have had pain.	
			En los ultimos 7 dias, tuve dolor	In the past 7 days, I had pain	
Itchy Skin	Skin Symptoms	Picazones	Picazón	Itching	Picazón
			Picazon la Piel	Itchy Skin	
Skin: In the past 7 days, I had (check all that apply)	Skin Symptoms	Piel: En los últimos 7 días, tuve…(marque toda que aplica)	… (marque todo lo que aplica)	…(check all that apply)	Piel: En los últimos 7 días, tuve…(marque todas que apliquen)
				… (all that apply)	
			…(todas que apliquen))		
I had tight/thick/tough skin or stiffness (fibrosis)	Skin Fibrosis Symptoms	Esta parte de mi cuerpo sintió aprieto, grueso, duro, o rígido (fibrosis)	Tuve piel estirada/gruesa/dura o rigidez (fibrosis)	I have stretched/thick/hard skin or stiffness (fibrosis)	Tuve sensacion de mi piel tirante/grueso/duro o rigido (fibrosis)
				Felt tight, thick, hard, or stiff (fibrosis)	
			Se sintio apretada, gruesa, dura, o rigida (fibrosis)		
				I had this part of my body that is tight, thick, hard, or stiff (fibrosis)	
			Tuve esta parte de mi cuerpo apretado, grueso, duro o rígido (fibrosis)		
Lost Vision	Eye Symptoms	Visión perdida	Pérdida de vision	Loss of vision	Perdida de vision
			Vista perdida	Lost sight	
Eye sensitivity to light	Eye Symptoms	Sensibilidad a lujo	Sensibilidad ocular a la luz	Eye sensitivity to light	Sensibilidad a la luz
			Sensibilidad a la luz	Sensitivity to light	
			Sensibilidad de vista a la luz	Sight sensitivity to light	
Mouth/Oral Cavity [Symptom]	Mouth Symptoms	Boca (exterior e interior):	Boca/cavidad bucal	Mouth/Oral Cavity	Boca/cavidad buccal
Teeth problems (cracking, tooth decay or cavities, loss of teeth)	Mouth Symptoms	Problemas con los dientes (rajar, dientes picados, perdió un(os) diente(s))	Problemas con los dientes (quebraduras, dientes picados o caries, perdida de dientes	Tooth problems (chipping, pitting or cavities, missing teeth)	Problemas con los dientes (diente rotos, picados, caries, perdida de diente(s)
				…loss of tooth (s)	
			…perdida de diente(s)	Tooth problems (chipped, pitted tooth, cavities, missing tooth (s)	
			Problemas con los dientes (diente rotos, picados, caries, perdida de diente(s)		
Nausea/vomiting	GI Symptoms	Nausea/vomitar	Nausea o vómito	Nausea or vomiting	Nausea/vomito
			Nausea/vomito	Nausea/vomiting	
			Nauseas y vomitos	Nausea and vomiting	
			Nausea/vomitos		
Voice: in the past 7 days, I had (check all that apply)	Voice Symptoms	Voz: En los últimos 7 días, tuve …(marque toda que aplica)	…(marque todo lo que aplica)	…(check all that apply)	Voz: En los últimos 7 días, tuve …(marque todas que apliquen)
				…(all that apply)	
			…(todas que apliquen)		
Difficulty or limitation in moving part(s) of my body	Movement Symptoms	En los últimos 7 días, tuve dificultades con o limitaciones en mover partes de mi cuerpo	En los últimos 7 días, tuve dificultad o limitaciones en mover partes de mi cuerpo	In the last 7 days, I had difficulty or limitations in moving parts of my body	En low ultimos 7 dias, tuve dificultades o limitaciones en mover partes de mi cuerpo
			Dificultades o limitaciones con el mover.	Difficulties or limitations with moving	
How would you rate your breathing discomfort right now? (0 = None, 10 = Unbearable)	Breathing Symptoms	Evalúa la calidad de sus respiraciones en este momento.	¿Cómo calificaría su dificultad para respirar en este momento?	How would you rate your shortness of breath right now?	¿Cómo calificaría su dificultad para respirar en este momento?
			marque el numero que represente la dificultad de su respiracion en este momento	Mark the number that represents the difficulty of your breathing at this moment	
Do your symptoms/current health situation negatively affect your normal social activities with family, friends, or neighbors?	Social Function Symptoms	¿Afectan a sus actividades sociales normales con la familia, amigos, o vecinos sus síntomas o estatus de salud?	¿Sus síntomas o su estado de salud actual le afectan o interfieren con sus actividades sociales regulares con familiares, amigos, o vecinos?	Do your symptoms or your current health condition affect or interfere with your regular social activities with family, friends, or neighbors?	¿Sus síntomas o su estado de salud actual le afectan o interfieren con sus actividades sociales regulares con familiares, amigos, o vecinos?
			Sus sintomas o estado de salud afectan de forma negativa sus actividades sociales con la familia, amigos, o vecinos		
				Your symptoms or health status negatively affect your social activities with family, friends, or neighbors	
			Sus síntomas o estatus de salud ha afectado a sus actividades sociales normales con su familia, amigos o vecinos?	Has your symptoms or health status affected your normal social activities with your family, friends or neighbors?	
In the past 7 days, while eating or swallowing, I sometimes…(check all that apply)	Swallowing Symptoms	En los últimos 7 días, mientras que estuve comiendo o tragando, a veces me…(marque todo que aplica)	En los últimos 7 días, mientras que comía o tragaba, a veces me…(marque todo lo que aplica)	In the last 7 days, while eating or swallowing, I sometimes…(check all that apply)	En los últimos 7 días, mientras que estuve comiendo or bebiendo en los ultimos 7 dias, a veces me…(marque todas que apliquen)

				While I've been eating or drinking in the last 7 days, I sometimes…(check all that apply)	
			Mientras que estuve comiendo or bebiendo en los ultimos 7 dias, a veces me…(marque todas que apliquen)		
				In the last 7 days, while eating or swallowing, sometimes	
			En los ultimos 7 dias, mientras comia o tragaba, a veces		

#### 3.2.2 Different words or grammatical structures representing different concepts or meanings

The cognitive interviews regarding the remaining 9 problematic items revealed that these items differed in words or grammatical structure and represented different concepts or meanings to the participants (See Table 3). The items primarily involved pain (3 of 9) and swallowing (3 of 9). Other symptoms were ear, movement, and breathing. For example, the term “stabbing” as a descriptor of pain had alternate translations of “puñalada,” “dolor punzante”, and “punzante.” The participants interpreted the items as “pain from a stabbing,” “stabbing pain,” and “throbbing pain.” From the cognitive interviews, it was unclear that a cross-cultural translation equivalent existed for the swallowing term “gagging.” Ultimately, the team decided to replace the term “gagging” with a field for patients to enter free text to describe their swallowing problems. The team discussed and refined all other items to account for cross-cultural variations and included the items in the final translation of the Spanish ePVA.

**Table 3:** Spanish ePVA items With different words or grammatical structures representing different concepts or meanings.

English	Item Description	Forward Translation	Suggested Alternate Translations By Expert Panel and Patient Participants	Back Translation	Final Version in Spanish ePVA
Tenderness	Pain Symptoms	Delicadeza	Ternura	Tenderness	Ternura
			Sensibilidad	Sensitivity	
			Dolor al tocar	Pain when touching	
Stabbing	Pain Symptoms	Puñalada	Dolor Punzante	Pulsating pain	Dolor Punzante
			Punzante	Throbbing pain	
Soreness	Pain Symptoms	Malestar general	Adolorido	Sore	Dolorido
			Dolorido	Sore	
Ringing In Ears (Tinnitus)	Ear Symptoms	Ruido en los ojos (tintineo)	Ruido en los oidos (tinnitus)	Noise in the ears (tinnitus)	Zumbido en los oídos (tintineo)
			Ruido o zumbido en los oídos	Noise or ringing in the ears	
			Zumbido en los oidos	Ringing in the ears	
In the past 7 days, I had difficulty breathing or shortness of breath	Breathing Symptoms	En los ultimos 7 dias, fue difícil respirar y/o sentía como tuve falta de aliento.	dificultad para respirar	Shortness of breath	En los ultimos 7 dias, fue difícil respirar y/o sentía como tuve falta de aire.
			Fue difícil respirar y/o me sentía corto(a) de aliento.	It was difficult to breathe and/or I felt short of breath	
			Sentia falta de respiracion		
				I felt short of breath	
			Tuve falta de aire	I felt short of breath	
Cough	Swallowing Symptoms	Tose	Tosia	Coughed	Da Tos
			Tos	Cough (Pushing air out mouth)	
			Toso		
				To push air out through mouth	
Choke	Swallowing Symptoms	Atraganta	Ahogaba	Drowning	Ahogo
			Ahogo	Drowned	
Gag	Swallowing Symptoms	Amordaza	Hacía por volver	To return	Item deleted because of lack of equivalent conceptual meaning across cultures
				Choking	
			Atraganto	Using your mouth to chew/having to do with your mouth	
			Amordaza		
[Difficulty] Bending Over	Movement Symptoms	Doblarse	Doblarme	I bend over	Doblarse
			Agachar	Crouch/duck	

## 4 Discussion

Nearly 46.2 million immigrants lived in the US in 2022, the highest number in the nation's history (53). Translating healthcare materials is critical to providing quality healthcare for those with LEP; however, translations may not account for structural and cultural differences in shared languages across countries, such as English, Spanish, Arabic, or Russian. This manuscript reports the use of CVI methodology, a comprehensive and systematic translation approach, to produce the Spanish ePVA PROM with acceptable cultural relevance and translation equivalence.

Traditionally, translating an English measure into another language consists of two independent translators agreeing on a standard version, which is then retranslated back into English (back translation) by two other translators in agreement on one version. These agreed-upon back translations may be evaluated by bilingual experts for their conceptual equivalence, use of everyday language, and clarity (54). Finally, the English back translation is compared with the original by one of the persons who developed the instrument.

However, these traditional methods may not capture the cultural variations within shared languages. Immigrant populations may speak common languages across countries, but the cultures and traditions of their ancestral country influence the grammatical structure and conceptual meanings. Methodologies that produce quality translations include processes that result in a questionnaire with acceptable cultural relevance and translation equivalence for use in multicultural populations with LEP (55).

### 4.1 The benefits and limitations of the CVI methodology

The CVI methodology incorporates the aforementioned steps and includes quantitative ratings of the cultural relevance and translation equivalence and qualitative comments by experts evaluating the translation. This approach identifies problematic items and informs additional research to refine the final translation, such as cognitive interviews. Accordingly, the benefit of the CVI methodology over traditional methods is that it reduces inherent bias by identifying problematic items that affect the translation's quality. The CVI methodology may require more resources than traditional methods, such as independent translators for the cognitive interviewing component. As artificial intelligence advances, machine translations of written documents may increase to preserve resources. The quality of machine translations has improved dramatically; nevertheless, these translations may require a native speaker to check to ensure their cultural relevance and translation equivalence (56). Researchers using machine translations will need to plan for these resources accordingly as part of the translation process.

### 4.2 Public health implications

This research has public health implications for patients with LEP. Notably, the 9 problematic items that differed in grammatical structure and conceptual meaning across Spanish-speaking cultures represented the critical symptoms of HNC: pain, swallowing, and breathing. A possible rationale for this finding is that symptoms represent an individual's perception of the biological activity of cancer and its treatment (57, 58), and are affected by psychosocial factors, including anxiety, depression, and patients' perception of vulnerability (57, 59, 60). Thus, capturing an individual's perception of symptoms may pose challenges in cross-cultural translations and hamper communication between patients and providers regarding the presence and severity of symptoms. Indeed, a retrospective study of patients with lung cancer found that Hispanic patients had a higher pain burden than White patients (61). Evolving research has also uncovered that symptom disparities may arise from the intersection of minority populations, medical conditions, and non-medical drivers of health (62). For example, researchers have found that the presence of a higher number of comorbid conditions, lower education levels, and lower income among these populations may drive pain disparities in minority populations (63, 64). Our research adds to this body of work by suggesting that cross-cultural differences in shared languages among patients with LEP may also contribute to symptom disparities across cancer types.

PROMs may help reduce these disparities in symptoms. Research shows that systematic use of PROMs during cancer care is associated with improved symptom control, decreased acute care use, and prolonged survival (6–8). Barriers to PROMs for patients with LEP include a lack of validated PROMs translated into non-English languages and insufficient time and resources to accommodate patients with LEP (65). The findings from this study underscore the need to translate PROMs into different languages using methodologies such as CVI that account for cultural differences in shared languages. Further research is needed to help clinicians and researchers better understand whether these structural and cultural differences in language contribute to symptom disparities.

### 4.3 Strengths and limitations of the research

This research study on using the CVI methodology to produce the Spanish ePVA has strengths and weaknesses. A strength is the recruitment of translators with origins in multiple countries and regions to provide a cross-cultural translation of the ePVA. The limitations include that the project was a single-site study at a research-intensive academic center in the northeastern US. Further, the population for the cognitive interviews did not include women despite the recruitment of all genders and races. Additionally, the CVI methodology is intended as a beginning step in developing measures and should always be followed with additional psychometric testing. With these limitations, the team may have missed key cultural nuances that could decrease the performance of the Spanish ePVA. To offset these limitations, the team has conducted follow-up beta testing of the Spanish ePVA with men and women and is planning for additional analyses, including additional psychometric testing to analyze the performance of the Spanish ePVA in studies with larger populations.

## 5 Conclusion

Translations of health materials may not account for structural and cultural differences in shared languages across countries, such as English, Spanish, Arabic, or Russian. This manuscript presents the use of the CVI methodology with cognitive interviews to translate the ePVA from English into Spanish, resulting in a PROM with acceptable cultural relevance and translation equivalence. The CVI approach may require additional resources but can produce measures that facilitate communication between the patient with LEP and providers, potentially decreasing symptom disparities in patients with LEP.

## Data Availability

The raw data supporting the conclusions of this article will be made available by the authors, without undue reservation.

## Supplementary material

The Supplementary Material for this article can be found online at: https://www.frontiersin.org/articles/10.3389/frhs.2025.1582127 /full#supplementary-material.
