# Supplementary material for: Using content validity index methodology for cross-cultural translation of a patient-reported outcome measure for head and neck cancer
Source: Front Health Serv. 2025 Jun 20;5:1582127. doi: 10.3389/frhs.2025.1582127 (PMC12226542; doi:10.3389/frhs.2025.1582127)
Supplement: Supplementary file 1 [file Table1.docx]

SUPPLEMENT A

COGNITIVE INTERVIEW PROBES

Below are cognitive interview questions for patients to help determine the important items to patients and whether the questions address symptoms that they are experiencing. In recognition that the cancer may affect communication, we will accept verbal, written, or typed answers in the event that someone has difficulty talking.

Q1.What does the following sentence mean to you? “En los ultimos 7 dias, tuve dolor”

Q2 What is the difference between these two phrases? “sensibilidad ocular a la luz” y “Sensibilidad a la luz”

Q5 What is the difference between these two phrases? “Dolor punzante” y “punzante”

Q6 What is the difference between these two phrases? “Dolor punzante” “Puñalada”

Q6 What is the difference between these two phrases? “Zumbido en los oídos (tintineo)” y ‘Ruido en los ojos (tintineo)”

Q8 . What does the following word mean to you? “tos”?

Q9 What is the difference between these two phrases? “Marque todas las que apliquen” y “marque todo lo que aplica” y “marque todo lo que aplique”

Q10 What is the difference between these two phrases? “En los últimos 7 días, esta parte de mi cuerpo sintió aprieto, grueso, duro, o rígido (fibrosis)” y “En los últimos 7 días, tuve piel estirada/gruesa/dura o rigidez (fibrosis)”

Q11 What does the following word mean to you “Picazón”?

Q12 What does the following word mean to you “Nausea/vomito” ?

Q13 What is the difference between these two phrases?? “En los últimos 7 días, fue difícil respirar y/o me sentía corto(a) de aliento.” y “**En los ultimos 7 dias, fue difícil respirar y/o sentía como tuve falta de aire”**

Q14 What do the following words mean to you “atraganto” “ahogo” ?

Q15 What is the difference between these two phrases? “¿Sus síntomas o su estado de salud actual le afectan o interfieren con sus actividades sociales regulares con familiares, amigos, o vecinos?.” y “¿Afectan a sus actividades sociales normales con la familia, amigos, o vecinos sus síntomas o estatus de salud?”

Q16 What does the following phrase mean to you? “Por favor toque las partes de la figura en la pantalla donde Ud. tiene dolor“?

Q17 What do the following words mean to you “Adolorido” Y “Malestar general “ ?

Q18 What does the following phrase mean to you? “Ojos llorosos”

Q19 What does the following phrase mean to you “Problemas con los dientes (quebraduras, dientes picados o caries, perdida de dientes”?

Q20 What is the difference between these two phrases?? “Dificultad en saborear la comida” y “Dificultades en saborear comida”

Q21 What does the following phrase mean to you? “Respuesta requerida al eligir esta opcion”

Q22 What does the following phrase mean to you? “En los últimos 7 días, tuve dificultad o limitaciones en mover partes de mi cuerpo”

Q23 What does the following phrase mean to you? “En los últimos 7 días, tuve dificultad o limitaciones en mover partes de mi cuerpo”

Q24 What does the following phrase mean to you? “Para elegir algo, toque las marcas o puntos.”

Q25 What does the following phrase mean to you? “¿Cómo calificaría su dificultad para respirar en este momento? (0=Normal/ninguna dificultad, 10 = Intolerable)”

Q26 What are the differences between these two phrases? “En los últimos 7 días, mientras que comía o tragaba, a veces me” y “En los últimos 7 días, mientras que estuve comiendo o tragando, a veces me”?

Q27 What does the following word mean to you “Amordaza” ?

Q28 What does the following phrase mean to you? “Por favor, explique - Note: Es requerido responder cuando elija esta opción.”

Q29 ¿What does this following phrase mean to you? “En los últimos 7 días, a veces me sentía ansioso(a).”

Q31 ¿What is the difference between these two words “doblarme” y “doblarse “?

Q32 What is the difference between these two phrases? “lado izquierda de la cara” y “Cara izquierda”

Q33 What is the difference between these two phrases? “pierna superior derecho” y “Muslo derecho”

Q34 What is the difference between these two phrases? “Pierna izquierda inferior” y “Muslo izquierdo”

Q35 What is the difference between these two phrases? “dolorido” y “Malestar general”?

Q36 What does the following phrase mean to you? “Visión nublada”

Q37 What does the following phrase mean to you? “Otro - Note: Es requerido responder cuando elija esta opción.”

Q38 What is the difference between the two phrases? “Dificultad para oír claramente” y “Dificultades en oír bien”

Q39 What does the following phrase mean to you? “Dificultad en oler”

Q40 What does the following phrase mean to you? “Saliva gruesa y pegajosa”

Q41 What does the following phrase mean to you? “Babear por la boca” y “Babear de la boca”

Q42 What does the following phrase mean to you? “Estreñimiento (Sentirse tapado)”

Q43 What is the difference between these two phrases? ”lado izquierdo del cuello” y “Cuello izquierdo

Q44 What does the following phrase mean to you? “Dificultades en saborear comida”
